# Induction of cellular prion protein (PrPc) under hypoxia inhibits apoptosis caused by TRAIL treatment

**DOI:** 10.18632/oncotarget.3028

**Published:** 2015-01-20

**Authors:** Jin-Young Park, Jae-Kyo Jeong, Ju-Hee Lee, Ji-Hong Moon, Sung-Wook Kim, You-Jin Lee, Sang-Youel Park

**Affiliations:** ^1^ Biosafety Research Institute, College of Veterinary Medicine, Chonbuk National University, Jeonju, Jeonbuk, South Korea; ^2^ Department of Bioactive Material Sciences and Research Center of Bioactive Materials, Chonbuk National University, Jeonju, Jeonbuk, South Korea

**Keywords:** PrPc, Hypoxia, TRAIL, HIF-1α, Colon cancer

## Abstract

Hypoxia decreases cytotoxic responses to tumor necrosis factor-related apoptosis-inducing ligand (TRAIL) protein. Cellular prion protein (PrPc) is regulated by HIF-1α in neurons. We hypothesized that PrPc is involved in hypoxia-mediated resistance to TRAIL-induced apoptosis. We found that hypoxia induced PrPc protein and inhibited TRAIL-induced apoptosis. Thus silencing of PrPc increased TRAIL-induced apoptosis under hypoxia. Overexpression of PrPc protein using an adenoviral vector inhibited TRAIL-induced apoptosis. In xenograft model *in vivo*, shPrPc transfected cells were more sensitive to TRAIL-induced apoptosis than in shMock transfected cells. Molecular chemo-therapy approaches based on the regulation of PrPc expression need to address anti-tumor function of TRAIL under hypoxia. Molecular chemo-therapy approaches based on the regulation of PrPc expression need to address anti-tumor function of TRAIL under hypoxia.

## INTRODUCTION

Tumor necrosis factor (TNF)-related apoptosis-inducing ligand (TRAIL), also known as the Apo2 ligand, is a membrane-bound cytokine. TRAIL induces apoptosis in a variety of cancer cell types [1–3] but most normal cells appear to be resistant to TRAIL activation [4–6]. TRAIL is a potent inducer of apoptosis in a wide variety of cancer cells, both *in vitro* and *in vivo*, without damage to normal tissues [3, 6, 7]. This unique selectivity for cancer cells has drawn considerable attention to TRAIL as a potential therapeutic modality to manage human cancers.

Hypoxic conditions elicit cellular responses that improve cell survival through adaptive processes [8, 9]. Particularly, biologically and therapeutically important hypoxia occurs in many solid tumor masses [9], as the center of rapidly growing solid tumors is easily exposed to hypoxic conditions [10]. This cell response to hypoxia is an adverse prognostic indicator in cancer, as it is associated with tumor progression and resistance to therapy [11–13]. Recent findings show that hypoxia increases the anti-apoptotic potential of tumor cells by regulating the molecules involved in apoptosis signaling pathways [14]. These effects of hypoxia render tumor cells resistant to various cancer therapies including TRAIL treatment and facilitate survival of tumor cells [12, 15].

Hypoxia-inducible factor-1alpha (HIF-1α) is a transcription factor that directly transactivates important genes for growth and metabolism of solid tumors [16–18]. Furthermore, these changes in gene expression allow solid tumors to utilize physiological responses to hypoxia; thus, improving their survival and metastasis [19, 20]. HIF-1α is overexpressed in cancer, its expression level is correlated with patient mortality [21, 22] and it plays an important role in human cancer cell invasion and metastasis [23, 24].

Cellular prion protein (PrPc) is the prion protein isoform found in normal tissues [25, 26]. PrPc is a copper-binding glycosylate-phosphatidylinositol-anchored membrane protein expressed predominantly in neurons and to a lesser degree in some extra-neuronal tissues, such as muscle, lymphoid tissue, and gastrointestinal mesenteric ganglion cells [27]. PrPc protects breast cancer cells from TNF-induced apoptosis [28]. Over-expression of PrPc converts TNF-sensitive MCF-7 cells into TNF-resistant cells by up-regulating cytochrome *c* release from mitochondria [29, 30]. Moreover, several studies have shown that PrPc increases immune-reactivity within neuronal processes in the ischemic penumbra and that hypoxic conditions induce overexpression of PrPc in gastric cancer cells [31]. However, it is unclear how TRAIL-induced apoptosis is resisted under hypoxic conditions, and the role of PrPc under hypoxic conditions remains unclear in human colon carcinoma cells.

In this study, we investigated whether silencing of PrPc protein under hypoxic condition would blocks inhibition of TRAIL-induced tumor cell apoptosis and PrPc overexpression is associated with resistance to TRAIL-induced apoptosis under hypoxic or normal oxygen conditions in colon cancer cells. Our results showed that overexpressed PrPc increased resistance to TRAIL-induced apoptosis under normal oxygen condition, whereas silencing of PrPc caused sensitization to TRAIL-induced apoptosis under hypoxic conditions.

## RESULTS

### Up-regulation of HIF-1α influences PrPc expression in HCT116 cells

**Figure 1:**
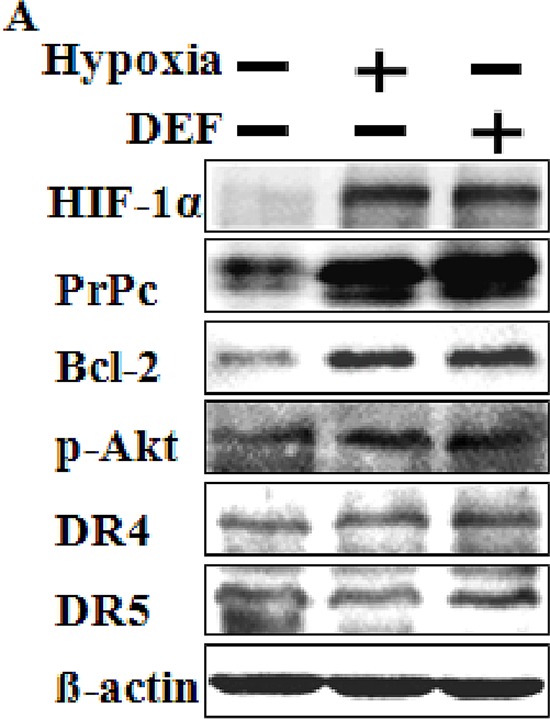
Hypoxia regulates HIF-1α and PrPc **(A)** Western blot analysis of HIF-1α, PrPc, Bcl-2, p-Akt, DR4 and DR5 from HCT116 cells pre-exposed to normoxia or hypoxia for 24 h and treated with or 10 μM DEF under normoxia for 24 h. β-actin was used as a loading control.

Previous reports have demonstrated that the expression of normal PrPc is regulated by HIF-1α, and that PrPc overexpression induced by hypoxia plays a pivotal role in hypoxic inhibition of PrPc-induced neuron cell death [32]. A Western blot analysis indicated that hypoxic conditions up-regulated the HIF-1α protein compared to that under normal oxygen condition (Figure 1A). Interestingly, PrPc expression in Western blots was similar to that of HIF-1α, as PrPc increased in DEF-treated cells and under hypoxic conditions. Moreover, the cell signaling-mediated proteins Bcl-2 and p-Akt increased expression under hypoxic conditions but DR4 and DR5 did not (Figure 1A). These results indicate that over expression of PrPC was related with HIF-1α, and as such, are possible mechanisms involved in the hypoxic conditions of HCT116 cells.

### Hypoxia suppresses TRAIL-induced apoptosis in human colon carcinoma cells

**Figure 2:**
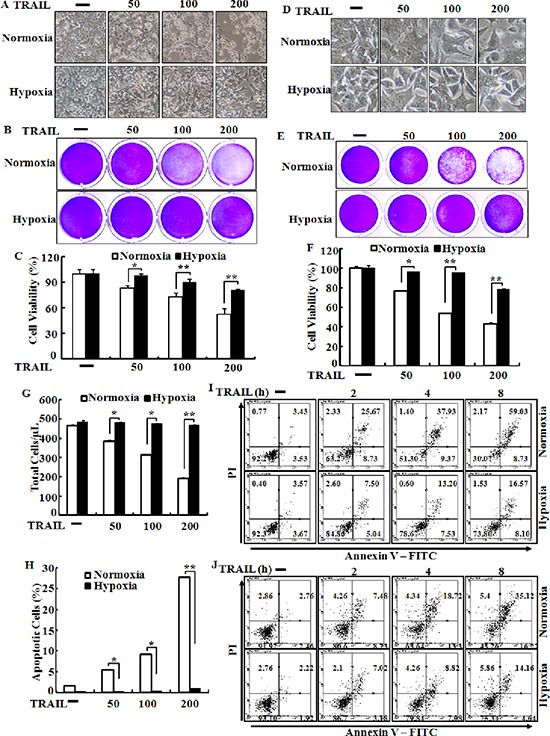
Hypoxia inhibits TRAIL-induced apoptosis human colon carcinoma cells HCT116 cells were pre-exposed to normoxia or hypoxia for 24 h and further incubated with recombinant TRAIL (0–200 ng/ml) for an additional 4 h under the same conditions. **(A)** Treated cells were photographed with a light microscope (× 200). **(B, C)** Viable cells were stained with crystal violet. Viability of control cells was set at 100%, and viability relative to the control was estimated. These results are representative of three independent experiments. **(D)** HCT8 cells were photographed with a light microscope (× 200). **(G, H)** Viable cells were stained with crystal violet. Viability of control cells was set at 100%, and viability relative to the control was estimated. These results are representative of two independent experiments. Bar graph indicates the number of total cells and percent of apoptotic cells. *p < 0.05 or **p < 0.01 significant differences between control and each treatment group. In (**I** and **J**) HCT-116 and HeLa cells were exposed to hypoxia 12 hr after then treated with 100 ng/ml TRAIL for indicated time periods, respectively. Cell viability was measured by the Annexin V assay.

**Figure 3:**
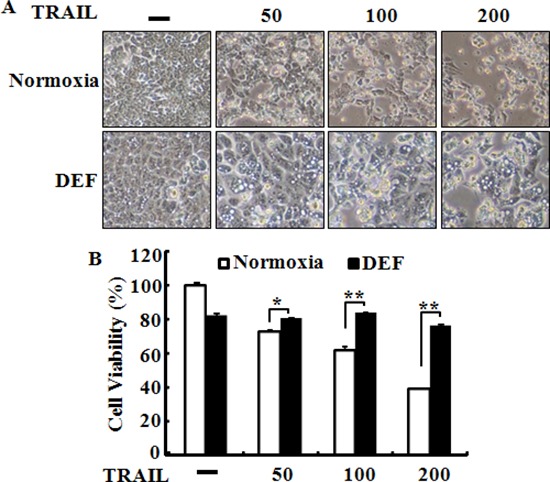
Deferoxamine inhibits TRAIL-induced apoptosis **(A)** HCT116 cells were pre-treated with 10 μM deferoxamine (DEF) under normoxia for 24 h before treatment with 0–200 ng/ml of TRAIL, for 4 h. Treated cells were photographed with a light microscope (× 200). **(B)** Cell viability was measured by the crystal violet staining method. Viability of control cells was set at 100%, and viability relative to the control is presented. The bar graph indicates the mean ± standard error of the mean (SEM) (*n* = 3). *p < 0.05 or **p < 0.01 significant differences between control and each treatment group.

Tumor cells exposed to hypoxia are resistant to TRAIL-induced apoptosis [15, 33]. To investigate the effects of hypoxia on TRAIL-induced apoptosis, HCT116, HCT8 and NCL-H747 cells were incubated under hypoxic or normal oxygen conditions and treated for 24 h with or without TRAIL (0–200 ng/ml) for 4 h (Figure 2). An examination of HCT116 and HCT8 cell morphology showed an inhibitory role of hypoxic conditions in TRAIL-induced apoptosis (Figure 2A and 2D). We further studied the sensitivity of HCT116 and HCT8 cells to TRAIL-mediated cell death using a crystal violet-based cell viability assay (Figure 2B and 2E), and determined that HCT116 and HCT8 cells were resistant to the cytotoxic action of various concentrations of TRAIL under hypoxic conditions compared with those under normal oxygen condition. The cells were responsive to TRAIL treatment (10–50% reduction in viability of all cell populations), but hypoxia had only a very slight effect on cell viability (Figure 2C and 2F). To confirm these results, we examined whether hypoxia influences TRAIL-induced apoptosis in NCL-H747 human colon cells under the same conditions (Figure 2G and 2H). Similar to HCT116 and HCT8 cells, these data show that hypoxia blocked TRAIL-induced apoptosis in NCL-H747 cells. Moreover, HCT116 and HeLa cells exposed to hypoxia were increased than that of control groups in a time-dependent manner (1–8 h) after treatment with TRAIL (Figure 2I and 2J) HCT116 cells were pre-incubated with a hypoxia mimic reagent, deferoxamine (DEF) alone for 24 h and treated with or without TRAIL for an additional 4 h. The data showed that DEF also had a protective effect on TRAIL-induced apoptosis in HCT116 cells (Figure 3A and 3B) as that of cell treated with hypoxia. Therefore, our data indicate that hypoxia inhibited TRAIL-induced apoptosis in human colon carcinoma cells.

### Knockdown of PrPc blocks protection of hypoxia in TRAIL-treated cells

**Figure 4:**
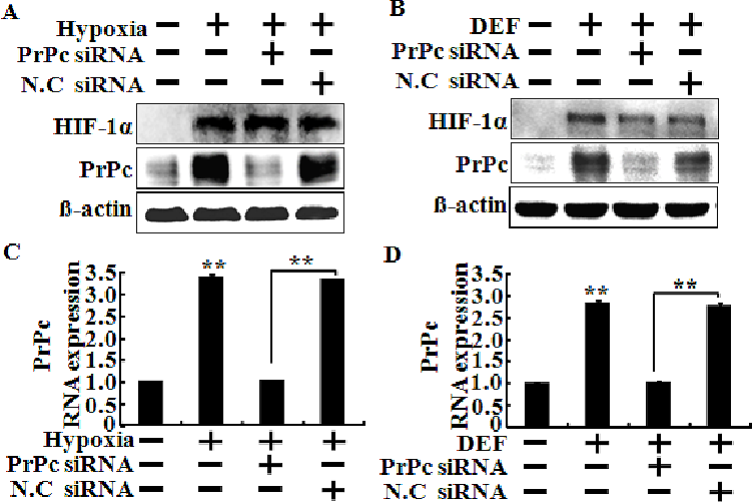
Knockdown of PrPc inhibited the HIF-1α-mediated PrPc expression **(A, B)** HCT116 cells were pre-treated 20 nM PrPc RNAi for 24 h and pre-exposed to DEF or hypoxia for 24 h and then detected the protein levels of HIF-1α, PrPc. β-actin was used as a loading control. **(C, D)** The mRNA levels were determined by quantitative real-time PCR for HIF-1α and PrPc. This *treatment* is as *described* in C. These results are representative of three independent experiments. **p < 0.01 compared to basal conditions.

To verify that the HIF-1α protein plays a protective role in hypoxic colon cancer cells via PrPc, we used PrPc siRNA to determine the effect of silencing PrPc in HCT116 cells (Figure 4). HCT116 cells were pre-treated with 20 nM PrPc siRNA for 24 h. A Western blot analysis showed PrPc protein was down-regulated by PrPc siRNA although, HIF-1α activation was up-regulated under hypoxic conditions or treatment of DEF (Figure 4A and 4B). RT-PCR analysis was used to determine the levels of HIF-1α and PrPc mRNA, and showed that PrPc mRNA levels were significantly lower in cells transfected with siRNA molecules than that in cells transfected with scrambled negative control siRNA (Figure 4C and 4D).

**Figure 5:**
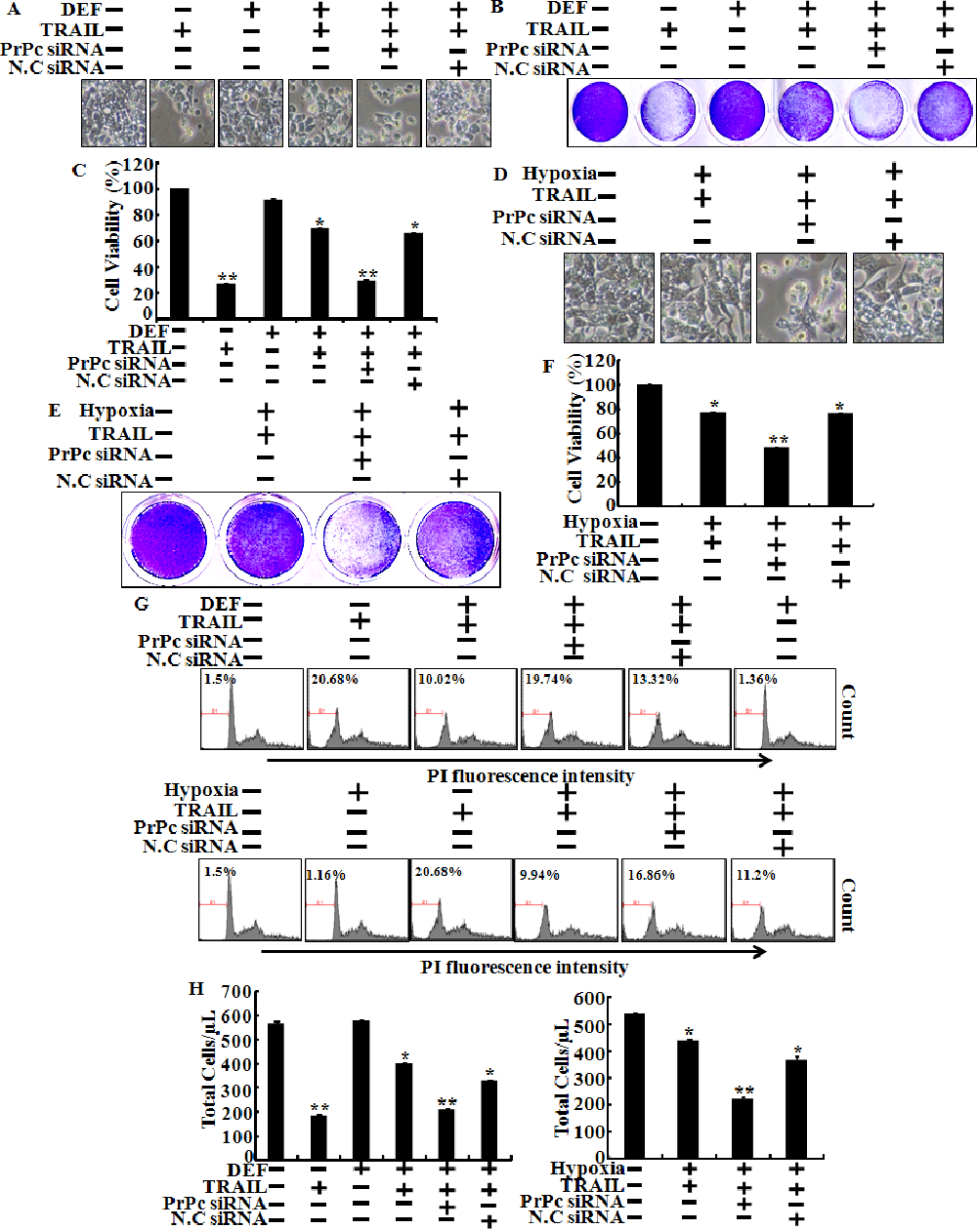
Knockdown of PrPc blocks protection of hypoxia in TRAIL-treated cells HCT116 cells were pre-treated 20 nM PrPc RNAi for 24 h and pre-exposed to DEF or hypoxia for 24 h and further incubated with recombinant TRAIL (200 ng/ml) for an additional 3 h under the same conditions. **(A, D)** Treated cells were photographed with a light microscope (× 200). **(B, C, E, F)** Viable cells were stained with crystal violet. Viability of control cells was set at 100%, and viability relative to the control was estimated. These results are representative of two independent experiments. **(G)** *Prnp* gene in HCT116 cells was knocked down (PrPc siRNA) using the PrPc siRNA oligomer and then exposed to hypoxic conditions with or without TRAIL 200 ng/ml for 24 h. Cell viability was measured by PI staining assay, **(H)** Bar graph indicates the number of total cells. HCT116 cells viability was measured by PI staining assay. Percent of numerical value represents the population of apoptotic cells. (E, F) Bar graph indicates the number of total cells and percent of apoptotic cells. *p < 0.05, **p < 0.01: compared to basal conditions.

**Figure 6:**
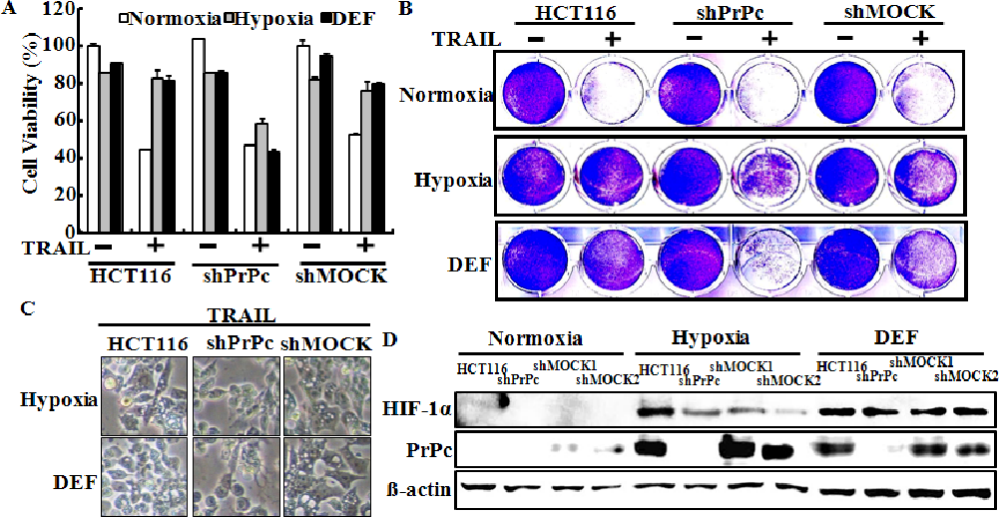
Lentiviral shRNA knock-down of PrPc sensitizes HCT116 cancer cells for TRAIL-induced colon cancer cell death **(A, B)** PrPc-shRNA or mock transfected HCT116 cells were cultured in 21% or 1% oxygen tension for 24 h or pre-treated with 10 μM of deferoxamine, and then treated with the indicated dose (200 ng/ml) of TRAIL for 3 h. Cell viability was measured by the crystal violet staining method. Viability of control cells was set at 100%, and viability relative to the control is presented. The bar graph indicates the mean ± S.E.M. (*n* = 3). **P < 0.01, significant differences between control and each treatment group. **(C)** The treated cells were photographed with a light microscope (× 200). **(D)** Western blot analysis of HIF-1α and PrPc from PrPc-shRNA HCT116 cells pre-exposed to normoxia or hypoxia for 24 h and treated with or 10 μM DEF under normoxia for 24 h. β-actin was used as a loading control.

Next to investigate whether inhibiting TRAIL-induced apoptosis is associated with the regulation of PrPc under hypoxic conditions, we analyzed the sensitivity of siRNA-transfected HCT116 cells to TRAIL-induced apoptosis under hypoxic conditions (Figure 5). Cells were pre-treated 20 nM PrPc siRNA for 24 h, pre-exposed to DEF or hypoxia for 24 h, and further incubated with recombinant TRAIL protein for an additional 3 h under the same conditions. Scrambled PrPc siRNA was used as the negative control. We studied the morphology of HCT116 cells (Figure 5A and 5D), as well as cell viability using the crystal violet assay (Figure 5B, 5C, 5D and 5F). We confirmed HCT116 cells viability by PI staining (Figure 5G). The bar graph indicates the number of cells under hypoxic conditions (Figure 5H). Knockdown of PrPc partly restored the sensitivity of HCT116 cells to the cytotoxic action of TRAIL in the HIF-1α activation system (Figure 5). PrPc down-regulation enhanced TRAIL-induced apoptosis under the HIF-1α activation system, resulting in apoptosis in almost 60% of the HCT116 cells (Figure 5C and 5F). These data demonstrated that hypoxic conditions up-regulate PrPc expression and protect against TRAIL-induced cell death. In contrast, silencing PrPc increased TRAIL-induced apoptosis and decreased resistance to TRAIL under hypoxic conditions. shPrPc transfected cells (shPrPc) increased TRAIL-mediated apoptosis compared to that of mock-transfected cells (shMock) cells under hypoxic conditions (Figure 6A–6C). Also, shMock cells increased PrPc and HIF-1α expression under hypoxia, whereas shPrPc transfected cells did not change the levels of HIF-1α (Figure 6D).

### Therapeutic effect of TRAIL in colon cancer HCT116 xenograft

**Figure 7:**
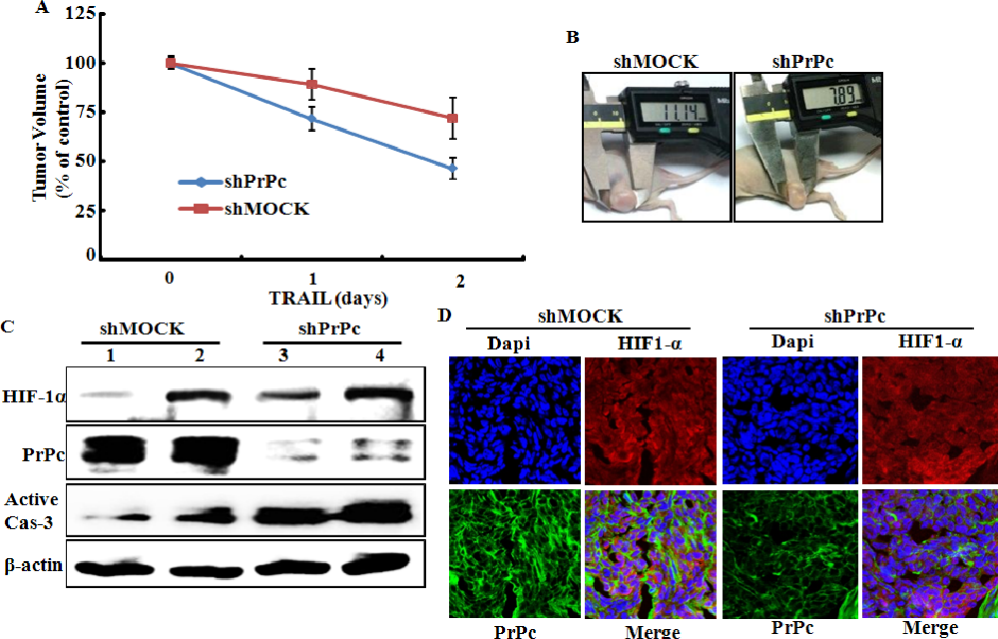
PrPc-shRNA HCT116 tumors increased TRAIL-mediated apoptosis in mouse xenograft **(A, B)** shPrPc HCT116 tumors growth in mouse thigh is suppressed by intra-tumoral injection of TRAIL in BALB/c nude mice when TRAIL treatment was started at tumors size were ≥ 150 mm^3^ after tumor implant. **(C)** Western blot analysis of HIF-1α, active-caspase-3 and PrPc from shPrPc HCT116 tumors in xenograft mice. Lane 1 : shMock1, Lane 2 : shMock2, Lane 3 : shPrPc1 Lane 4 : shPrPc2. **(D)** Representative images of PrPc and HIF-1α expression in shPrPc HCT116 tumors described in (C)

We determined the role of HIF-1α-mediated PrPc on the TRAIL-mediated apoptotic response observed in sh-mock or shPrPc transfected HCT116 human colon cancer cells using a xenograft model following intra-tumoral injection of TRAIL or PBS. Nude mice with a shPrPc and shMock HCT 116 xenograft were treated with TRAIL for 24 h, 20 days after tumor implantation. Decreased tumor progression was observed in shPrPc HCT116 xenograft mice compared to that in shMock HCT116 xenograft mice after a subcutaneous injection of 250 ng/g TRAIL (Figure 7A and 7B). We next examined the relationship between HIF-1α and PrPc expression in HCT116 xenograft mice. Western blot analysis showed that shPrPc HCT116 xenograft mice showed increased active-caspase-3 expression compared to that in shMock HCT116 xenograft mice (Figure 7C). Also, in the xenograft mice models, PrPc knock-out was not influenced to the expression of HIF-1α (Figure 7C). The immunochemistry results showed that the central part of the HCT116 xenograft had increased HIF-1α expression but that of the shPrPc HCT116 xenograft showed decreased PrPc expression. These results indicate that HIF-1α-mediated resistance of TRAIL-mediated toxicity is related to PrPc expression, and as such, are possible mechanisms involved in the hypoxic conditions of colon cancer.

### PrPc overexpression inhibits TRAIL-induced apoptosis under normal oxygen condition

**Figure 8:**
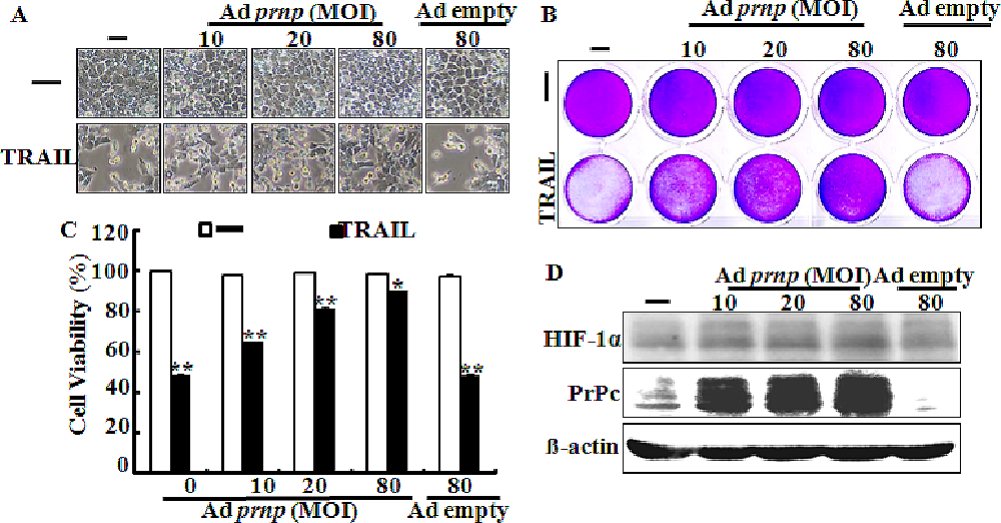
PrPc overexpression inhibits TRAIL-induced apoptosis Transfection of Ad-*Prnp* at multiplicity of infection (MOI) 0, 10, 20, 80with Ad-empty at multiplicity of infection 80 for 24 hand incubated with 200 ng/ml TRAIL for 3 h. **(A)** Treated cells were photographed with a light microscope (× 200). **(B, C)** Viable cells were stained with crystal violet. Viability of control cells was set at 100%, and viability relative to the control was estimated. These results are representative of two independent experiments. **(D)** Western blot analysis indicated that overexpression of PrPc was investigated in transfected HCT116 cells after 24 h using the specific anti-PrPc antibody 3F4. *p < 0.05, **p < 0.01 : compared to basal conditions.

Our data show that silencing PrPc enhanced TRAIL-induced apoptosis under hypoxic conditions. To verify that PrPc has protective effects in TRAIL-related cytotoxicity, the recombinant adenovirus-expressing full length *Prnp* gene (Ad-*Prnp*) was utilized to overexpress the *Prnp* gene in PrPc knockdown HCT116 cells under normal oxygen condition (Figure 8). Transfection of HCT116 cells with Ad-*Prnp* resulted in overexpression of PrPc compared with that in Ad-empty transfected cells under normal oxygen condition. We transfected Ad-*Prnp* at a multiplicity of infection (MOI) of 0, 10, 20, or 80 or transfected with Ad-empty at MOI of 80 for 24 h with 200 ng/ml TRAIL for 3 h. An examination of HCT116 cell morphology showed that the Ad-Prnp transfected cells inhibited TRAIL-induced apoptosis under normal oxygen condition compared to that in Ad-empty transfected cells (Figure 8A). The crystal violet-based cell viability assay showed similar results (Figure 8B and 8C). In short, transfection of Ad-*Prnp* cells inhibited TRAIL-induced apoptosis, but cells transfected with Ad-empty cells did not. A Western blot analysis indicated that PrPc was overexpressed in transfected HCT116 cells after 24 h using the specific anti-PrPc antibody 3F4. The HIF-1α protein was not expressed under normal oxygen condition. The PrPc protein clearly increased in an MOI dependent manner following Ad-*Prnp* treatment (Figure 8D). These data indicate that inducing PrPc expression played a protective role against TRAIL-induced human colon carcinoma cell death.

## DISCUSSION

In this study, we demonstrated that hypoxia-related up-regulation of PrPc protein inhibited TRAIL-mediated apoptosis and PrPc protein expression related to hypoxic conditions stabilized HIF-1α in colon cancer cells. Solid tumors rapidly outgrow their blood supply, and the center part of a solid tumor has a lower oxygen concentration than that in the outer part. This hypoxic state brings about up-regulation of genes that confer resistance to chemotherapeutic drugs and radiotherapy and plays a major role initiating angiogenesis [34]. HIF-1α is a major factor involved in tumor progression including angiogenesis and metastasis by regulating mitogen activated protein kinases under hypoxic conditions [31, 35]. Liang et al. suggested that hypoxic conditions result in a time-dependent increase in PrPc expression in gastric cancer cells, and that up-regulation of PrPc decreased reduced following treatment with the extracellular regulated kinase inhibitor PD98059 [31]. Thus, we hypothesized that some transcriptional factors including HIF-1α regulate PrPc gene expression in colon carcinoma cells during hypoxia. PrPc gene and protein expression levels were increased by up-regulating HIF-1α in HCT116 cells, independent of oxygen concentration (Figures 1 and 3). However, HIF-1α expression remained unchanged following knockdown of PrPc expression (Figure 3A–3D). The shPrPc HCT116 xenograft model also showed that the inner part of the xenograft had increased HIF-1α expression compared with that of the outer part, independent of PrPc expression knockdown (Figure 7C and 7D). Thus, we suggest that up-regulating HIF-1α protein may modulate PrPc expression in rapidly growing colon cancers. In this study, we examined whether HIF-1α directly transcribe the PrPc mRNA by using EMSA assay and CHIP assay (data not shown), but the results did not show any significant results which indicating HIF-1α induces PrPc protein by binding to the promoter region of prion protein gene.

Some studies have suggested that up-regulating PrPc inhibits the effect of cancer chemotherapy [36–38]. One paper showed that treatment of PrP antibody inhibits the colon cancer cell growth [39]. Also, Meslin et al. showed that PrPc expression is regulated by Bcl-2 expression in human breast cancer cells and suggested that depleting PrPc facilitates Bax activity by inhibiting Bcl-2 expression, thereby increasing sensitization of breast cancer cells to TRAIL treatment [38]. PrPc-transfected cells showed increased expression of p-Akt, suggesting a possible novel mechanism by which a PI3K/AKT/PrPc mechanism regulates cancer cell survival [37]. We found that p-Akt and the Bcl-2 protein were up-regulated under hypoxic conditions (Figure 1). The xenograft model revealed that cleaved caspase-3 expression increased in shPrPc HCT116 xenograft mice compared to that in shMock HCT116 xenograft mice (Figure 7C). However, DR4/DR5 expression under hypoxic conditions did not affect regulation of DR4/DR5 (Figure 1). These results suggest that regulation of PrPc by HIF-1α is not associated with regulation of the death receptors during TRAIL-induced apoptosis.

TRAIL is a main factor in the innate immune response against tumor proliferation and development by binding to the DR4 and DR5 death receptors [40, 41]. The recent study suggested that HIF-1α may regulate decoy receptor expression, which plays a main role in impaired TRAIL-induced apoptosis under hypoxia [41]. Our data show that DEF-treated cells or cells exposed to hypoxia inhibited TRAIL-induced apoptosis in various colon cancer cell lines including HCT116, HCT8, and NCL-H747 (Figures 2 and 3). However, this inhibitory effect of tumor hypoxia was blocked by PrPc knockdown independent of HIF-1α expression (Figures 5 and 6). In the shPrPc HCT116 xenograft models, TRAIL treatment led to a significant decrease in tumor volume associated with lower PrPc expression, even though the expression of HIF-1α increased in the xenograft tumor mass, indicating that silencing of PrPc protein is an efficient strategy to inhibit colon cancer development (Figure 7). In addition, overexpression of the PrPc inhibited TRAIL-induced HCT116 cell death under normal oxygen condition (Figure 8), suggesting that normal prion protein may have a pivotal role in resistance to anti-cancer therapy under the hypoxic or normal oxygen condition.

Tumor radiation therapy is inhibited by hypoxia through Fas or TNF-α signals, which are related to hypoxia [42, 43]. A large body of clinical evidence suggests that tumor hypoxia negatively impacts radiotherapy, so there much research has been conducted into novel methods of improving tumor oxygenation, targeting hypoxic tumor cells, and modulating the response of hypoxic tumors to radiation [44, 45]. As more has been learned about the many ways hypoxia affects tumors, our understanding of the mechanisms connecting hypoxia to radio-sensitivity has become increasingly broad and complex [45, 46]. This has opened up new potential avenues for interrupting the negative effects of hypoxia on tumor radio-sensitivity. If we assume that HIF-1α controls PrPc, it is expected that therapeutic control under hypoxia may be related to by PrPc as well.

In conclusion, down-regulation of PrPc increased TRAIL-induced apoptosis under hypoxic conditions and PrPc was regulated by HIF-1α. We demonstrated that resistance to TRAIL under hypoxic conditions was regulated by PrPc. These findings strongly suggest that down-regulation of PrPc is sufficient to sensitize solid tumors to TRAIL-induced apoptosis.

## MATERIALS AND METHODS

### Cell culture

The human colon carcinoma cell lines HCT116, HCT8, NCL-H747 and human cervix adenocarcinoma cell line HeLa were maintained in RPMI1640 medium containing 10% fetal bovine serum (FBS; Invitrogen-Gibco, Carlsbad, CA, USA) and 100 μg·ml^−1^ penicillin-streptomycin in a humidified incubator maintained at 37°C and 5% CO_2_.

### Hypoxia treatment

The cells were exposed to hypoxia for 24 h, as described previously [47]. A hypoxia chamber was used to create a low oxygen environment; a gas mixture of 1% O_2_, 5% CO_2_, and 94% N_2_ flowed into the sealed chamber. Ambient air was evacuated through an outlet tube, and O_2_ flowed through the chamber for 2–3 min to maintain the desired O_2_ tension. The hypoxia-mimetic agent deferoxamine (DEF; 10 μM, Sigma-Aldrich, St. Louis, MO, USA) was used.

### Cell viability

HCT116 cells plated in 12-well plates were pre-exposed to normal oxygen or hypoxic conditions for the indicated times and were further treated with soluble recombinant human TRAIL protein (0–200 ng/ml) for 4 h [15]. Cell morphology was photographed under a microscope, and cell viability was determined by crystal violet staining, as described previously [32]. Cell viability was calculated from relative dye intensity and compared to that of controls.

### Quantitative real-time polymerase chain reaction (qRT-PCR)

Total RNA was extracted from HCT116 cells using the Easy-spin™ Total RNA Extraction kit (iNtRON Biotechnology, Daejeon, Korea). cDNA was synthesized using the TaKaRa Prime Script™ 1st strand cDNA synthesis kit (Takara Bio Inc., Shiga, Japan) following the manufacturer's instructions. The following primers were designed:

HIF-1α: forward 5′-CGCAAGTCCTCA AAGCAC AG-3′; reverse 5′-TGGTAGTGGTGGCATTAGCA-3′;

Prnp: forward, 5′-GTGCACGACTGCGTCAAT-3′; reverse, 5′-CCTTCCTCATCCCACTATCA-3′;

β-actin: forward, 5′-GCAAGCAGGAGTATGACG AG-3′; reverse, 5′-CAAATAAAGCCATGCCAATC-3′.

All Thunderbire™ SYBR qPCR mix reactions (TOYOBO, Tokyo, Japan) were performed on the CFX96 real-time PCR detection system (Bio-Rad, Hercules, CA, USA).

### Western blot analysis

The proteins were resolved by 10–15% sodium dodecyl sulfate-polyacrylamide gel electrophoresis and transferred to nitrocellulose membranes and were analyzed by Western blotting, as described previously [48]. The immunoblotting antibodies were HIF-1α (BD Biosciences, San Diego, CA, USA), PrPc (Millipore, Milford, MA, USA), Bcl-2 (Santa Cruz Biotechnology, Santa Cruz, CA, USA), phosphorylated-Akt (p-Akt) (Cell Signaling Technology, Danvers, MA, USA), DR4, DR5, and β-actin (Santa Cruz Biotechnology).

### Annexin V assay

Apoptosis was assessed by Annexin V assay in the detached cells using an annexin V assay kit (Santa Cruz Biotechnology, CA, USA) according to the protocol of manufacture. Annexin V measurement was determined by measuring the fluorescence at excitation 488 nm and emission 525/30 using a Guava EasyCyte HT (Millipore). Also, cell number was determined by flow cytometry.

### Immunofluorescence staining

The HIF-1α and PrPc expressions of xenograft examinations of tumor tissues were performed by immunofluorescence staining. Anti-HIF-1α (BD Biosciences) and anti-PrPc (Millipore), which is a mouse polyclonal antibody against channel isoform diluted 1:200. Obtain cryostat sections and put onto slide glass (Nalgen International, Glostrup, Denmark), washed in sterilized PBST for 10 min, blocked for 15 min with 5% FBS in PBST, incubated overnight at 4°C with primary antibodies, and diluted with 5% FBS in PBST. Alexa Fluor 488-labeled donkey anti-rabbit IgG antibody diluted 1:1000 (Molecular Probes, Sunnyvale, Ca, USA) was used to visualize channel expression by fluorescence microscopy.

### RNA interference

HCT116 cells were transfected with PrPc small interfering RNA (siRNA; Stealth RNAi, Santa Cruz Biotechnology) using Lipofectamine 2000, according to the manufacturer's instructions. The cells were plated in 24-well plates, pre-treated with 20 nM PrPc siRNA for 24 h, pre-exposed to DEF or hypoxia for 24 h, and incubated with recombinant TRAIL (0–200 ng/ml) for an additional 3 h under the same conditions. Scrambled PrPc siRNA (Invitrogen) was used as the negative control.

### Adenoviral infection

PrPc expressing adenoviruses (Ad) and Lac Z-bearing adenovirus in HCT116 cells have been described previously [49]. Cells were plated at 5 × 10^4^ cells per well in 12-well plates for transfection. The adenoviral vector (Ad-PrPc) was added to the HCT116 cell cultures in serum-free Opti-MEM for Prnp gene transduction (Gibco BRL). The cells were incubated for 4 h at 37°C in 5% CO_2_, washed twice with PBS, and fresh media containing 10% FBS was added. Cell structure was photographed under a phase-contrast light microscope after 24 h.

### Construction of the PrPc shRNA plasmid

shRNA against the PrPc gene was purchased from Santa Cruz Biotechnology. HCT116 cells were transfected with shPrPc, and stable transfectants were selected in puromycin after a 24 h recovery in standard growth medium. HCT116 cells transfected with a mock vector were used as a control.

### Xenograft model assay

BALB/c nude mice (CAnN.Cg-Foxn1nu/CrljOri) obtained from *ORIENT BIO INC (Seoul, Korea)* were used for the tumor xenograft studies. Tumors were implanted subcutaneously except for the HCT116 human colon cancer cells, which were implanted in the thigh. The size of the subcutaneously implanted tumors was measured at specified times, and TRAIL treatment started when tumors were ≥ 150 mm^3^. Prolonged experiments were carried out when the treated animals clearly reached tumor remission, so that any cure could be determined.

### Animals

All experiments received approval from the Chonbuk University Laboratory Animal Research Center. In total, 22 female 6-week-old BALB/c nude mice (CAnN.Cg-Foxn1nu/CrljOri), weighing 20–22 g, were obtained from *ORIENT BIO INC*. The animal room was maintained at 23 ± 1°C with alternating 12-h light and dark cycles. After a 1-week acclimatization period, the 22 mice were divided randomly into two groups. All animal procedures were performed in accordance with the institutional guidelines for the Chonbuk University Laboratory Animal Research Center.

### Statistical evaluation

All data are expressed as means ± standard deviations and compared using Student's *t*-test, analysis of variance, and Duncan's test with SAS statistical software (SAS Institute, Cary, NC, USA). Results were considered significant at *p* < 0.05.
